# Sperm DNA Methylation Epimutation Biomarkers for Male Infertility and FSH Therapeutic Responsiveness

**DOI:** 10.1038/s41598-019-52903-1

**Published:** 2019-11-14

**Authors:** Saturnino Luján, Ettore Caroppo, Craig Niederberger, Joan-Carles Arce, Ingrid Sadler-Riggleman, Daniel Beck, Eric Nilsson, Michael K. Skinner

**Affiliations:** 1 Urology Department, Hospital Universitari i Politècnic La Fe & Instituto de Investigación Sanitaria La Fe, Valencia, Spain; 2 Department of Maternal and Child Health, Reproductive and IVF Unit, Asl Bari, Conversano, Italy; 3 Department of Urology, University of Illinois Chicago, Chicago, IL USA; 4 Ferring Pharmaceuticals, 100 Interpace Parkway, Parsippany, NJ 07054 USA; 5 Center for Reproductive Biology, School of Biological Sciences, Washington State University, Pullman, WA 99164-4236 USA

**Keywords:** Ecology, Evolution, Systems biology, Molecular biology, Epigenetics

## Abstract

Male factor infertility is increasing and recognized as playing a key role in reproductive health and disease. The current primary diagnostic approach is to assess sperm quality associated with reduced sperm number and motility, which has been historically of limited success in separating fertile from infertile males. The current study was designed to develop a molecular analysis to identify male idiopathic infertility using genome wide alterations in sperm DNA methylation. A signature of differential DNA methylation regions (DMRs) was identified to be associated with male idiopathic infertility patients. A promising therapeutic treatment of male infertility is the use of follicle stimulating hormone (FSH) analogs which improved sperm numbers and motility in a sub-population of infertility patients. The current study also identified genome-wide DMRs that were associated with the patients that were responsive to FSH therapy versus those that were non-responsive. This novel use of epigenetic biomarkers to identify responsive versus non-responsive patient populations is anticipated to dramatically improve clinical trials and facilitate therapeutic treatment of male infertility patients. The use of epigenetic biomarkers for disease and therapeutic responsiveness is anticipated to be applicable for other medical conditions.

## Introduction

Human male sperm counts have dramatically declined over the past seventy years^1^. Recently, a thorough epidemiology study has demonstrated a 50% decline in human sperm numbers and quality in the last 50 years^2^. This correlates with a corresponding rise in male infertility which impacts the majority of the male population. Seminal parameters are historically the primary method used to identify male factor infertility^3^. This dramatic decline in human male sperm numbers and increase in male factor infertility is a significant economic and societal burden that predicts a future decreased fertility and is associated with a number of other disease etiologies^4^.

The primary source of this increased male factor infertility and decline in seminal parameters appear to be environmental exposures^2,4^. This includes a variety of toxicants, endocrine disruptors, abnormal nutrition, smoking, alcohol, and stress^2,5^. Animal models have demonstrated the direct actions of a number of environmental toxicants to reduce sperm numbers and promote testis disease and male infertility^5–7^. Various human male exposures also have been shown to associate with poor sperm parameters and male infertility^8,9^. The primary molecular actions considered involve environmental epigenetics.

Epigenetics is defined as “molecular factors or processes around DNA that regulate germline activity independent of DNA sequence and are mitotically stable”^5^. One of the principal epigenetic processes involved in sperm abnormalities is DNA methylation. Cytosine methylation at CpG sites can alter gene expression, and within sperm these sites are associated with reduced fertility and promotion of disease in offspring^5,6^. Altered sperm DNA methylation has been shown to be a biomarker for environmental exposures that associate with various pathologies later in life^5^. Although altered histone retention following protamine replacement in sperm and non-coding RNAs have also been shown to associate with male infertility^10–12^, the primary epigenetic biomarker investigated in the current study involves DNA methylation.

Animal models initially demonstrated a correlation with sperm DNA methylation and male infertility^6^. Human studies have also demonstrated a decreased fecundity associated with sperm DNA methylation alterations^13,14^. A sperm DNA methylation biomarker assay has been developed and validated, which uses a microarray approach to assess CpG islands within the genome^15^. Although this analysis only investigates approximately 1% of the genome, it has been shown to be useful in analysis of sperm DNA methylation in a clinical setting^15^. Subsequently, studies with *in vitro* fertilization (IVF) applications have used measurement of DNA methylation with this biomarker analysis to assess male infertility prior to assisted reproduction^16–19^. Since this previous analysis only examined a limited amount of the genome (i.e. <1%), the current study was designed to investigate a more genome-wide approach using low density CpG regions (i.e. 95% genome) to examine alterations in sperm DNA methylation.

A promising strategy to medically address male factor infertility involves the use of a follicle stimulating hormone (FSH) therapeutic treatment to potentially restore seminal parameters and reproductive capacity of the patient^20^. For example, observations suggest a beneficial effect of FSH treatment on spontaneous pregnancy and live birth rate in men with idiopathic male factor infertility^21^. Such treatments have also been used to potentially obtain better IVF outcomes in pregnancy and implantation rates. Although some male patients respond to this therapy, many do not, which limits the efficacy of the FSH treatment. The current study was designed to determine if an altered DNA methylation pattern (i.e. signature) in sperm may identify a biomarker for responsiveness to FSH treatment. Such an epigenetic biomarker could significantly improve the success of treatment options for male infertility. The ability to develop and use epigenetic diagnostics for pathology assessment and subsequent pharmaceutical drug responsiveness to FSH therapy may significantly impact our management of male infertility, as well as provide the proof of concept for other medical applications in the future.

## Results

The male idiopathic infertility and fertile (control) groups were recruited and patient sperm samples were collected at the Andrology Laboratory of Hospital Universitari i Politècnic La Fe, 46026 Valencia, Spain. An initial sperm sample was collected upon enrollment, a second at the start of treatment, and a third after three months of treatment. Twenty-one patients were enrolled which included nine patients in the fertile control group and twelve in the idiopathic infertility treatment group. Exclusion criteria included history of varicocele, cryptorchidism, hyperprolactinemia, malignant or benign tumors, known chromosomal abnormalities, testicular torsion or trauma, orchiditis, smoking, use of anabolic steroids, recreational drugs, body mass index >30 kg/m^2^, or intake of over 21 units of alcohol/week in the past 120 days. Therefore, only idiopathic male infertility patients participated in the study. The differences (mean ± SD) between the seminal sample and hormonal parameters of both groups are shown in Table 1. Semen samples with a period of sexual abstinence of 2–5 days were obtained and used for performing a spermiogram according to WHO (World Health Organization) 2010 guidelines. Hormone profile was dosed and analyzed following our clinical protocol in patients with male infertility. Results from the baseline variables from the group of fertile subjects and those with infertility showed that there is a statistically significant difference in sperm number (i.e. concentration) between the fertile group and the infertile group, with the latter having the lowest values (95% CI −83, −2.87), p < 0.001. Infertility patient samples also have a lower percentage of sperm motility, 95% CI [−2.62, 1.58], and p < 0.001. The control group (fertile) showed lower FSH levels than the infertility group, 95% CI [0.20, 0.95], p = 0.005. Although not statistically significant, basal estradiol levels are higher in the group of subjects with infertility, 95% CI [−0.03, 0.89], p = 0.06. Regarding the results in the infertility group after three months of FSH treatment (150 IU dose of FSH therapeutic three times per week), there was an increase in FSH levels after treatment, although not statistically significant, p = 0.063. However, the estimated confidence interval 95% difference should not be underestimated, 95% CI [−0.02, 0.73]. There was no statistically significant difference in regards to the group mean ± SD in the other variables analyzed before and after treatment. In terms of pregnancy rate, there were three pregnancies (3/10, 30%). Two occurred after ICSI procedures, one was spontaneous, and seven non-pregnancies (7/10, 70%). There are two patients pending of ICSI procedure with frozen samples. It is not possible to perform the statistical analysis on pregnancy rates due to the small number of events, and therefore only descriptive data can be obtained.

**Table 1:** Mean hormone and semen parameters at baseline and after three months.

Variable	Fertility Control baseline n = 9	Infertility Treatment baseline n = 12	Fertility Control 3 months n = 9	Infertility Treatment 3 months n = 12
	Mean (±SD)	Mean (±SD)	Mean (±SD)	Mean (±SD)
	Median (1st, 3rd Q.)	Median (1st, 3rd Q.)	Median (1st, 3rd Q.)	Median (1st, 3rd Q.)
Age (years)	39.11 (3.02)	35.83 (4.15)	39.11 (3.02)	35.83 (4.24)
	38 (37, 40)	35.5 (33.75, 37.25)	38 (37, 40)	35.5 (33.75, 37.3)
Seminal vol (mL)	3.12 (1.59)	2.73 (1.39)	2.82 (1.71)	2.93 (1.22)
	2.1 (2, 4)	3 (1.8, 4)	2 (2, 3)	3 (2, 3)
Sperm concentration (million/mL)	70 (37.39)	3.03 (2.49)	79.44 (54.85)	5.59 (6.71)
	50 (43, 111.3)	2 (1, 4)	55 (40, 100)	2.5 (0.88, 10.25)
Motility (%)	61.34 (20.98)	13.12 (8.27)	47.22 (10.03)	13.95 (10.39)
	55 (45.8, 67)	12.5 (5, 20)	45 (40, 60)	12.5 (5.7, 20)
Immotility (%)	38.66 (20.98)	86.88 (8.27)	52.78 (10.03)	86.05 (10.39)
	45 (33, 54.2)	87.5 (80, 95)	55 (40, 60)	87.5 (80, 94.3)
FSH (IU/mL)	3.01 (0.7)	5.79 (2.64)	3.33 (1.16)	7.97 (3.18)
	3.1 (2.5, 3.4)	5.5 (3.6, 7.67)	2.9 (2.6, 4.1)	7.75 (5.67, 8.8)
LH (IU/mL)	4.92 (2.23)	4.79 (2.43)	4.81 (1.12)	4.58 (2.24)
	4.6 (4.3, 6.1)	4.3 (2.67, 6.85)	4.9 (4.1, 5.3)	4.35 (2.77, 5.35)
Estradiol (pg/mL)	18.67 (8.46)	29.25 (13.89)	20.89 (10.13)	26.25 (8.47)
	16 (12, 23)	25 (19, 37)	21 (16, 28)	26.5 (17.75, 32.3)
Total Testosterone (ng/mL)	4.99 (1.4)	4.9 (1.45)	4.99 (1.61)	5.01 (1.49)
	4.75 (4.2, 5.7)	5.06 (3.88, 5.76)	4.7 (3.84, 6.3)	5.57 (4.19, 5.84)
Bioavailable testosterone (ng/mL)	2.13 (0.53)	2.47 (0.84)	2.08 (0.5)	2.32 (0.64)
	2.06 (1.9, 2.2)	2.46 (1.98, 2.77)	1.9 (1.75, 2.1)	2.41 (1.8, 2.71)

Hormone, semen and sperm parameters. The mean ± SD values for age (years), seminal volume (mL), sperm concentration (million/mL), motility (%), immotility (%), FSH (IU/mL), LH (IU/mL), estradiol (pg/mL), and testosterone (ng/mL). The fertile control and infertile treatment for baseline and after 3 months is presented with n-value indicated for each. In order to compare both groups, a numerical descriptive analysis has been made using the mean with standard deviation and the median (1st and 3rd quartile). The baseline differences between the treatment group and the control were then compared, as well as the effect of FSH between before and after treatment in the treated group. For this, mixed linear regression models were used in the case several measures per patient (semen volume and sperm concentration), and in the case of motility a beta logistic regression model was performed given its percentage character. The mixed models control the non-independence of data given that there are several measures per patient.

**Figure 1:**
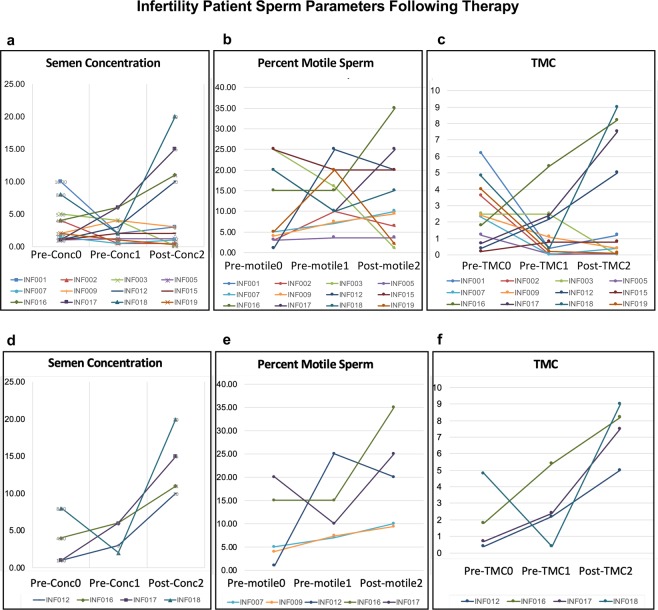
Infertility patients’ semen and sperm parameters upon recruitment (Pre-Conc 0) prior to FSH therapeutic treatment (Pre-Conc 1) and after 3 months of treatment (Post-Conc 2) for individual patients listed (color designated). Sample analyses for all patients are presented in **(a)** Semen concentration, **(b)** Percent motility sperm, and **(c)** Total motility count (TMC) (semen volume x concentration x motility). Infertility patients responding with >2-fold change following treatment are presented, **(d)** Semen concentration, **(e)** Percent motility sperm, and **(f)** TMC. The y-axis is magnitude of change between collections.

Individual patient information was used to identify the infertility patient responsiveness or non-responsiveness to FSH therapy, Supplementary Table S1. The infertility patients that showed a 2–3 fold increase in sperm number (semen concentration) and/or motility following three month treatment are shown in Fig. 1d–f and were designated as responders. Although some variation occurred from the initial sperm sample collected at enrollment and second sample at the start of the FSH treatment, the final values following treatment were generally higher for all parameters in responder patients, Fig. 1. The patients that responded to FSH therapy (Fig. 1c–e) were compared to the non-responsive patients (Fig. 1a–c) with the epigenetic analysis.

**Figure 2:**
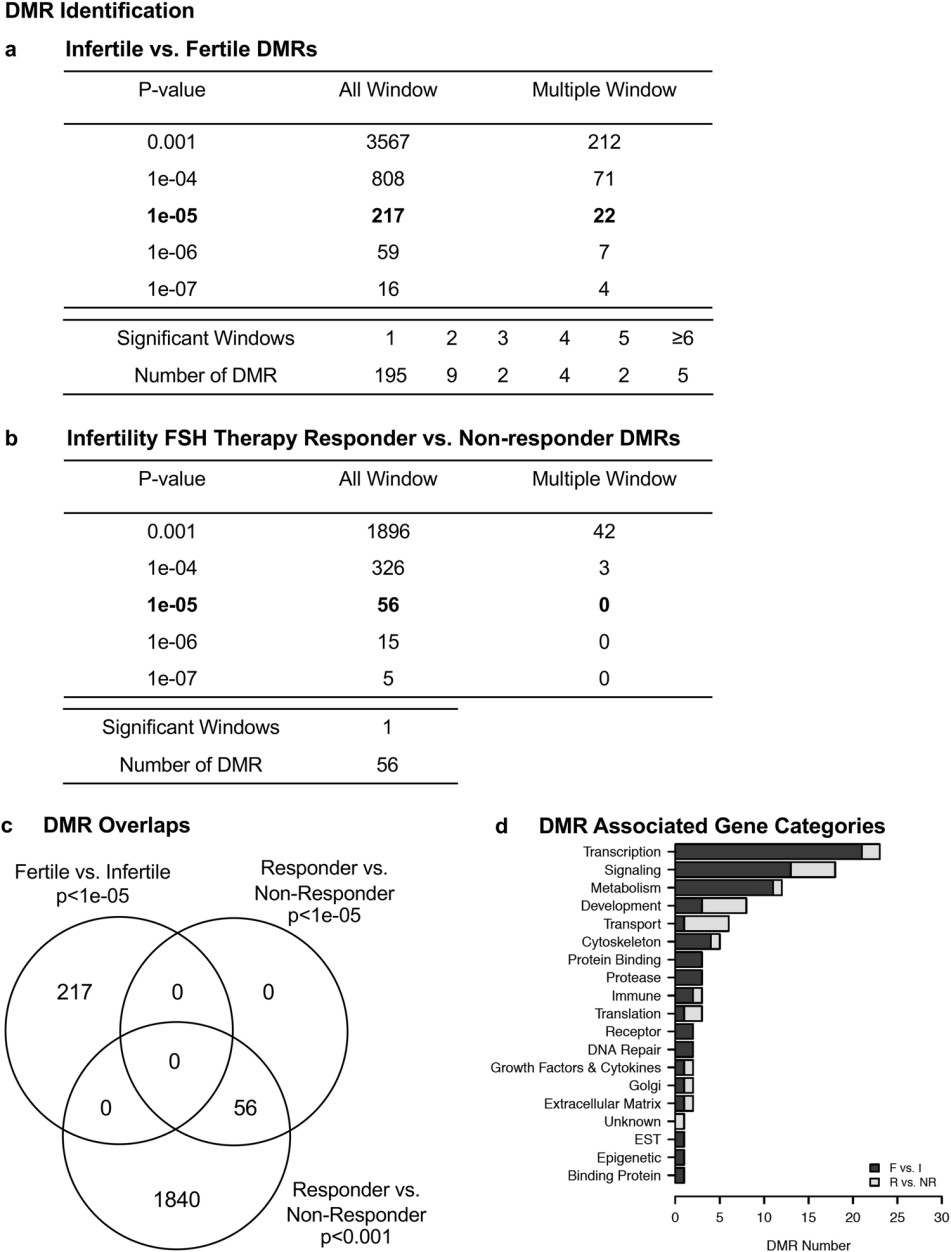
DMR identifications. **(a)** Fertility vs Infertility Sperm DMR Analysis. The number of DMRs found using different p-value cutoff thresholds. The all window column shows all DMRs. The multiple window column shows the number of DMRs containing at least two adjacent significant windows and the number of DMRs with each specific number of significant windows at a p-value threshold of 1e-05. **(b)** Infertility patient responder vs non-responder sperm DMRs. The number of DMRs found using different p-value cutoff thresholds. The all window column shows all DMRs. The multiple window column shows the number of DMRs containing at least two significant windows. The number of DMRs with each specific number of significant windows at a p-value threshold of 1e-05. **(c)** Venn diagram DMR signature for fertile vs infertile p < 1e-05 and DMR signature responder vs. non-responder at p < 1e-05 and p < 0.001. **(d)** DMR associated gene categories.

Individual patient samples from the initial sperm sample collected upon enrollment, the sample at the start of the FSH therapy treatment, and the sample after 3 months of treatment were prepared for semen analysis and select sets used for epigenetic analysis. The DNA was extracted from the sperm then fragmented for a methylated DNA immunoprecipitation (MeDIP) analysis in order to identify differential DNA methylated regions (DMRs). The MeDIP is a genome-wide analysis examining 95% of the genome comprising low density CpG regions in comparison to the less than 5% of the genome of high density regions and CpG islands. The MeDIP DNA is then prepared for next generation DNA sequencing and bioinformatic analyses, as described in the Methods section. A comparison of the sequences derived from fertile versus infertile patient sperm identified DMRs for infertility assessment, Fig. 2a. At a p-value of p < 1e-05 there were 217 DMRs identified, and the majority of these were within one 1000 bp windows with fewer having multiple 1000 bp window involved. The DMRs at a number of different p-values are presented, but the p < 1e-05 was used for subsequent data analysis and a list of these DMRs are presented with various genomic features in Supplementary Table S2. Therefore, a male infertility DMR signature was identified when comparing fertile versus infertile patients’ sperm DNA.

All the infertility patients had a sperm collection prior to a three month FSH therapeutic treatment period after which another sperm sample was collected for analysis. A comparison of sperm from the infertility patients who responded to FSH treatment versus those who did not respond identified DMRs associated with the responder patients, Fig. 2b. A variety of p-value DMR data is shown, and at p < 1e-05 there were 56 DMRs selected for subsequent data analysis. All the 56 DMRs had a single 1000 bp window that was statistically significant (p < 1e-05; FDR-adjusted p < 0.1). A list of the responder DMRs and genomic features is presented in Supplementary Table S3. An overlap of the responder DMRs with the infertility DMRs demonstrated no overlap at p < 1e-05, Fig. 2c. The overlap using a p < 0.001 for the responder DMRs also shows no overlap with the infertility DMRs suggesting distinct epigenetic biomarkers. Approximately 50% of the DMRs have associated genes within 10 kb of a gene and these are listed in Supplementary Tables S2 and S3. The gene categories of these DMR associated genes are summarized in Fig. 2d. Interestingly, the major categories of transcription, signaling, metabolism, transport and cytoskeleton are common between the infertility DMRs and responder DMRs. Therefore, an FSH therapeutic responder epigenetic biomarker (i.e. DMR signature) was identified when comparing infertility patient responder versus non-responder sperm. However, an expanded analysis with a greater n-value is needed to validate this therapeutic responder epigenetic biomarker.

**Figure 3:**
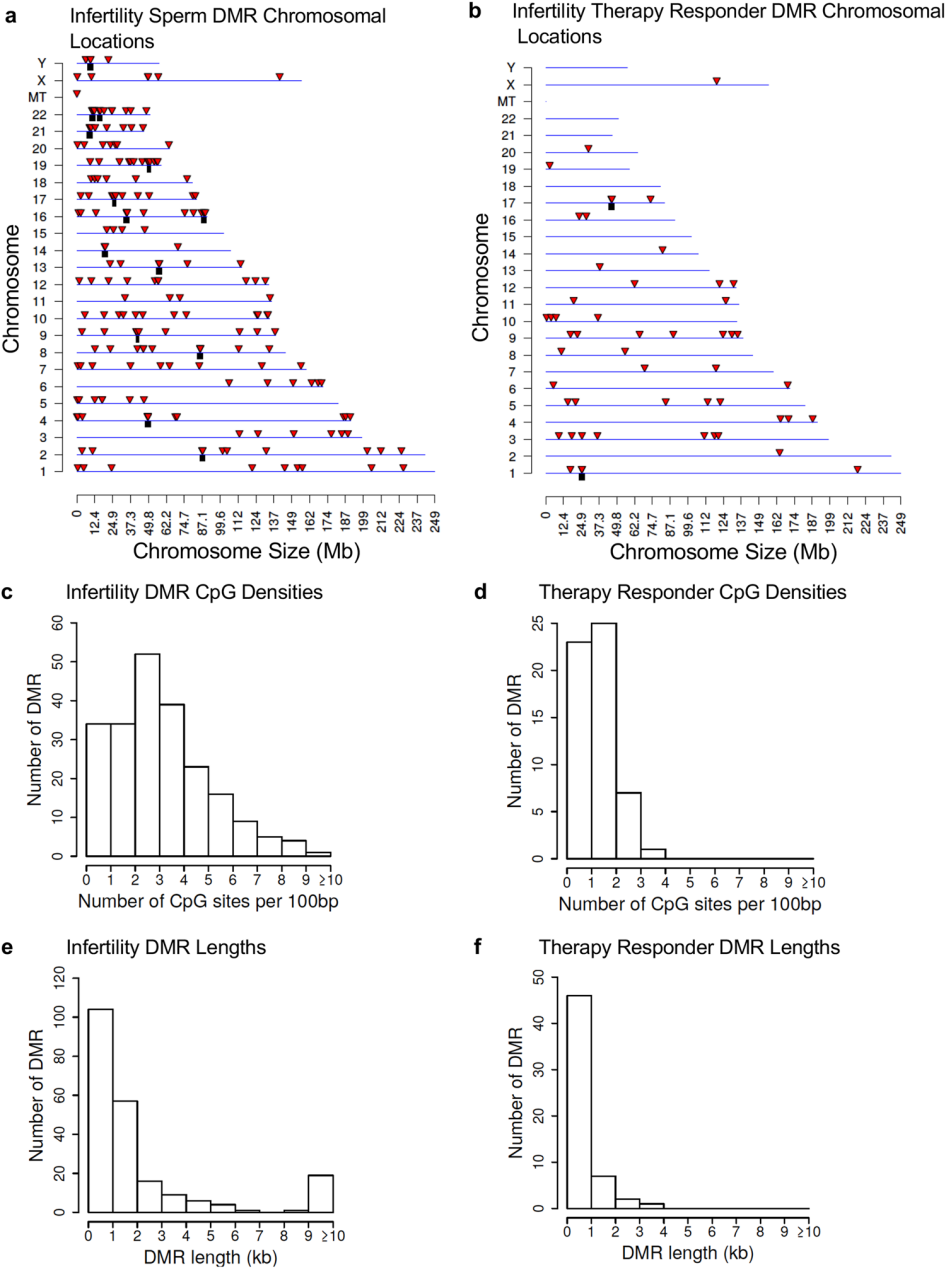
DMR genomic characteristics. **(a)** Chromosomal Locations of Fertility vs Infertility DMR Analysis. The DMR locations on the individual chromosomes. All DMRs at a p-value threshold of p < 1e-05 are shown with the red arrowhead and clusters of DMRs with the black boxes. **(b)** Responder DMR Signature Chromosomal Locations. The DMR locations (red arrowhead) and clusters of DMRs (black box) on the individual chromosomes. All DMRs at a p-value threshold of p < 1e-05 are shown. **(c)** DMR CpG density in the Fertility vs Infertility DMRs. The number of DMRs at different CpG densities. All DMRs at a p-value threshold of p < 1e-05 are shown. **(d)** The Responder signature DMR CpG density (number per 100 bp). The number of DMRs at different CpG densities are presented. All DMRs at a p-value threshold of 1e-05 are shown. **(e)** Fertility vs Infertility DMR lengths in kilobases. All DMRs at a p-value threshold of 1e-05 are shown. **(f)** The Responder signature DMRs size in kilobases. All DMRs at a p-value threshold of 1e-05 are shown.

The genomic features of the infertility DMRs and FSH therapeutic responder DMRs were investigated. The chromosomal locations of the DMRs within the human genome are presented in Fig. 3a,b. The red arrowhead indicates an individual DMR and the black box represents a cluster of DMRs. The infertility DMRs are present on all the chromosomes and mitochondrial DNA. The therapy responsiveness DMRs are also on most chromosomes. The CpG density where DNA methylation occurs is generally less than 10 CpG per 100 bp with 1–4 CpG predominant for the infertility and therapy response DMRs, Fig. 3c,d. The size of the DMRs was predominantly 1–4 kb for the infertility DMRs and 1–2 kb for the therapy response DMRs, Fig. 3e,f. Additional genomic features are presented in Supplementary Tables S2 and S3, which indicates approximately 90% of the infertility DMRs and 50% of the responder DMRs have an increase in DNA methylation and the rest a decrease in DNA methylation. Therefore, the majority of DMRs in infertility involve an increase in DNA methylation, while only half in the responder DMRs.

**Figure 4:**
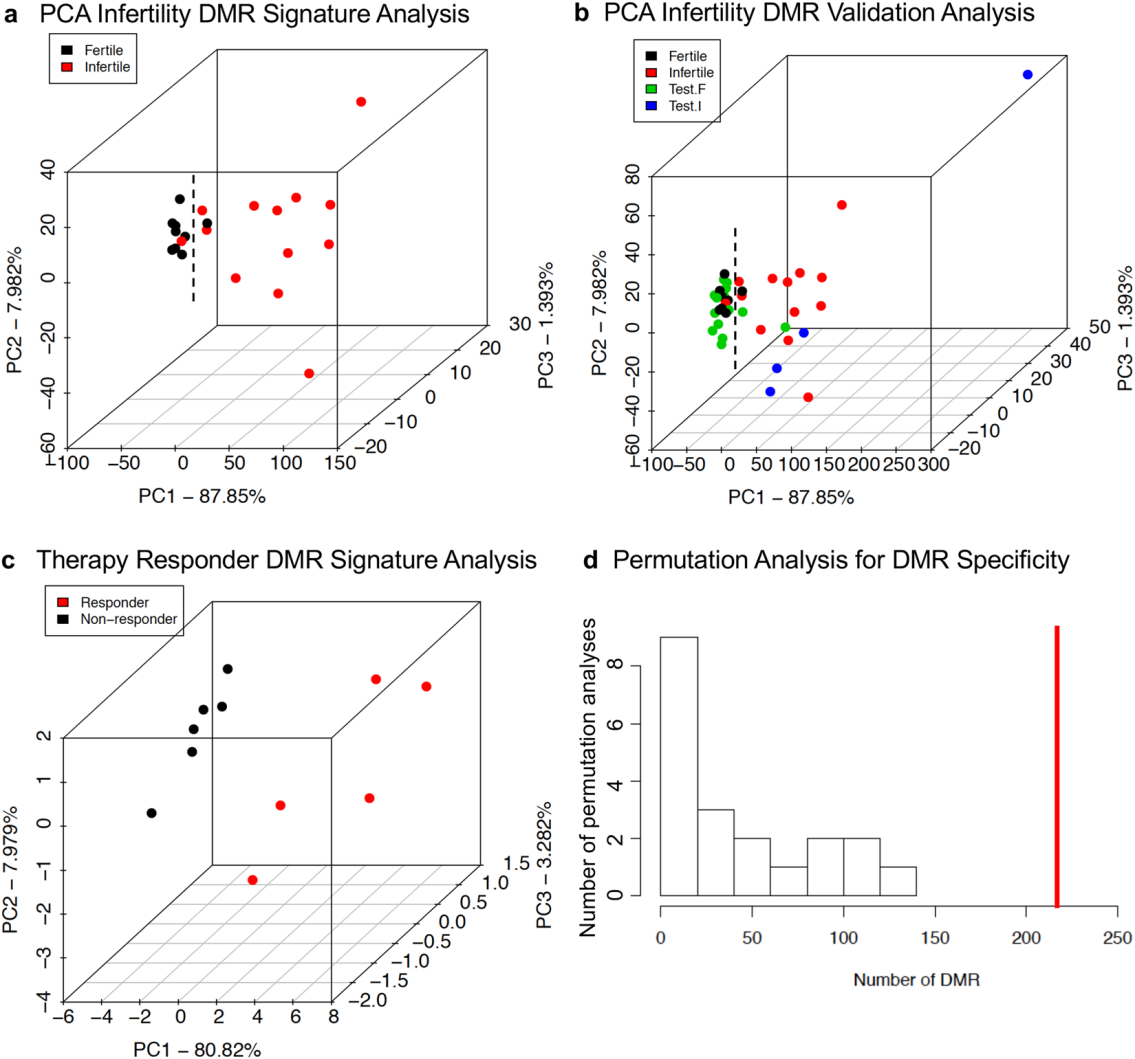
Principal component analysis. **(a)** Fertility vs Infertility DMR Principal Component Analysis for Individuals. The samples are plotted by the first three principal components. The underlying data is the RPKM read depth for the DMRs. The color code is listed for the fertile and infertile patients. **(b)** Fertility vs Infertility DMR Principal Component Analysis for Individuals. The samples are plotted by the first three principal components. The underlying data is the RPKM read depth for the DMRs. The color code is listed for the fertile and infertile patients. Selection failure correlations for fertile and infertile patients not used to generate the epigenetic signature. PCA Infertile vs Fertile p < 10^−5^. **(c)** Responder and non-responder PCA analysis for DMRs at p < 1e-05. The first three principal components used and samples color code index indicated. The underlying data is the RPKM read depth for all DMRs. **(d)** The number of DMR for fertility versus infertility comparison for all permutation analyses. The vertical red line shows the number of DMR found in the original analysis. All DMRs are defined using an edgeR p-value threshold of p < 1e-05.

The final analysis investigates the statistical significance and associations of the DMRs for each comparison and some initial validation analysis. A principal component analysis (PCA) of the infertility versus fertility DMR principal components is presented in Fig. 4a. There was a general clustering of the fertile DMRs and infertile DMRs from each other with only one DMR from each group outside the cluster. Therefore, good separation of the DMR in the PCA analysis was observed for the infertile versus fertile DMR groups. A validation set of samples were collected that were selection failures due to a variety of reasons and not used in the infertility DMR analysis for DMR identification, Fig. 4a and Supplementary Table S1. However, the sperm samples collected were used to determine fertility and infertility parameters. These selection failure samples were used as a validation test set of samples and analyzed with the MeDIP-Seq procedure. These were included in a separate PCA analysis. The test infertility samples clustered with the infertility group, and majority of the test fertility samples clustered with the fertility group, Fig. 4b. Two of the test fertility samples clustered with the infertility group. A PCA analysis with this validation set demonstrates the green DMR fertile test set primarily associates with the fertility patients while all the blue DMR infertile test set samples associate with the infertile group. This initial test set helps validate the infertility DMR signature identified in the current study. A similar PCA analysis of the FSH therapeutic responsiveness DMRs was performed. A clustering of the non-responsive DMRs was observed and all were distinct from the responsive cluster, Fig. 4c. No validation test set existed for the responsive DMR signature. A final permutation analysis was performed on the fertility versus infertility data to demonstrate the DMRs were not due to background variation and randomly generated. Insufficient data did not allow a similar analysis with the responder data sets. The permutation analysis demonstrates that the number of infertility DMRs generated from the comparison was significantly greater than the DMRs generated from random subsets within the analysis, Fig. 4d. The red line to the right indicates the comparison DMRs versus the low numbers from the random subset comparison. Although significantly more validation with larger clinical test sets is needed for both the infertility DMR biomarkers and FSH therapeutic responder DMR biomarkers, the current study provides the proof of concept that epigenetic biomarkers potentially exist and may be used in the diagnosis and potential treatment of male idiopathic infertility.

## Discussion

Male infertility over the past century has been observed to have dramatically declined^1,2^, with recent analysis of data for the past 50 years noting a 50% reduction in male sperm counts^2^. The primary causal factors suggested are environmental exposures influencing testis biology and sperm production^2–9^. In rodent models a number of defined toxicants and other exposures promote testis effects associated with a reduction in sperm number^5–7^. The current estimated infertility range is approximately 15–20% of the human male population^2^. A common strategy for medically assisted reproduction when male factor infertility is identified involves *in vitro* fertilization and intracytoplasmic sperm injection (ICSI), which are invasive and expensive procedures^22^. In addition to low sperm counts associated with infertility, there is also an increase in idiopathic infertility, which can have normal sperm cohorts and motility^2,22^. While seminal parameters are commonly used to screen for male factor infertility, the sperm number, motility and shape cannot fully explain the infertility^2,22^. The development of a clinical diagnostic analysis based on molecular alterations in the sperm would help address this clinical problem.

A promising approach for the clinical therapy of male infertility is the use of endocrine therapeutics, similar to what is used in the female^23^. Therapy with exogenous FSH is achieved by administration of urinary or recombinant FSH preparations or human menopausal gonadotropin (hMG) preparations, with the latter providing both FSH activity and luteinizing hormone (LH) activity. In women, FSH therapy is successfully used to stimulate oogenesis, and a similar approach would be expected to induce spermatogenesis^20^. Due to the variable response within the infertile population, a diagnostic test to assess a responder versus non-responder individuals would be expected to significantly enhance the utility of FSH therapeutics.

All clinical therapeutic studies have identified responder and non-responder subpopulations. Those that are efficacious for the majority of the population are generally not as concerned with the non-responder population. When the majority of the disease population does not respond, such as immune therapy for arthritis^24^, the advancement of a molecular diagnostic for the responder versus the non-responder population would be very useful in the management of the disease. Although a number of disease biomarkers or diagnostics have been identified for disease, few have been observed for specific responder versus non-responder subpopulations.

The current study was designed to identify a molecular biomarker or diagnostic for male idiopathic infertility and provide the proof of concept that an epigenetic analysis will be useful. Previously, the laboratory of Dr. Doug Carrell has utilized an analysis for DNA methylation using a microarray of CpG islands and methylation sites constituting a couple percent of the human genome to identify altered methylation in sperm from infertility patients^15–17^. These previous observations support the potential use of an epigenetic diagnostic for male infertility patients. Observations are expanded in the current study with a genome-wide analysis that constitutes 95% of the human genome and advanced molecular analysis.

Observations from the current study demonstrate a genome-wide analysis of DNA methylation identifies a male infertility signature of DMRs that are present in male infertility patients. There was an efficient separation between the fertile versus infertile patient population with minimal overlap. A validation with a test set of infertile and fertile patients, not used in the initial establishment of the infertility DMRs, also efficiently separated the infertile versus the fertile patients. The infertility signature of DMRs was found in all the infertile patients’ sperm samples showing the efficiency of the molecular biomarkers. Interestingly, the majority of the DNA methylation change involved an increase in DNA methylation (i.e. hypermethylation), which suggests during early gametogenesis and/or spermatogenesis development of the sperm a hypermethylation may be an aspect of the male infertility molecular disease etiology. Further analysis of this phenomenon may help elucidate the molecular basis of how male infertility develops. The development of a male infertility diagnostic will be useful for the clinical management of the male infertility patient. Due to the increasing male infertility in the human population over the past fifty years, a greater demand for such an analysis in an assisted reproduction setting such as an IVF clinic is anticipated.

Observations also demonstrate that an epigenetic DNA methylation biomarker can be developed to identify pharmaceutical responders versus non-responders to FSH treatment among male infertility patients. The infertility responder versus non-responder DMR signature identified efficiently distinguished the two populations, and in contrast to the infertility diagnostic, the responder DMR signature involved an equal distribution of hypermethylation (increase) and hypomethylation (decrease) changes. No overlap was observed between the infertility DMRs and responder DMRs, suggesting a distinct set of epigenetic alterations. Clearly an expanded clinical trial is needed to improve and validate the responder versus non-responder diagnostic. However, this is one of the first proof of concept that a therapeutic epigenetic biomarker could be developed. The current FSH therapeutic preparations in combination with the advancement of this responder diagnostic could allow for more effective patient management for infertility. In addition, this molecular approach to assess patient responder versus non-responder will assist in better drug development and design in the future.

## Conclusions

In conclusion, the current study identified a male infertility epigenetic DMR signature for use as a diagnostic, as well as an FSH therapy response diagnostic within this patient population. The advancement of such technology is anticipated to enhance the diagnosis and management of male infertility patients, as well as improve general therapeutic options and therapeutic development. This provides the proof of concept that epigenetic diagnostics can be developed and applied to pathologies and disease. Few applications of such epigenetic analysis have been used for disease diagnosis and therapy, but the current study suggests such applications are feasible. Expanded clinical trials are now needed to help validate and apply this novel technology to the management and treatment of male infertility.

## Material and Methods

### Clinical sample collection and analysis

A single center (Urology Department at Hospital Universitari i Politècnic La Fe), prospective and open clinical study. The IRB approval code protocol 2015-002521-19. We included two groups (infertility vs fertility). History of varicocele, cryptorchidism, hyperprolactinemia, malignant or benign tumors, known chromosomal abnormalities, testicular torsion, testicular trauma, orchitis, smoking, use of anabolic steroids,recreational drugs, body mass index >30 kg/m^2^, or Intake of over 21 units of alcohol/week in the past 120 days, were all used as exclusion criteria, such that all cases were idiopathic male infertility. The infertility men (inability of the couple to become pregnant after one year of sexual activity), included caucasians between 25–45 years of age with a total sperm concentration (concentration in millions/mL x volume in mL) between 1–10 million (oligozoospermia) in at least 2 spermiograms obtained after a 2–4 day period of sexual abstinence and with a 7-day separation period between tests. Semen samples with a period of sexual abstinence of 2–5 days were obtained and used for performing a spermiogram according to WHO 2010 guidelines. The hormone profile used inclusion criteria of FSH 2–12 IU/mL, total testosterone >300 ng/mL and bioavailable testosterone (calculated with the Sexual Hormone Binding Globulin or SHBG albumin) >145 ng/dL. Blood collections were directly obtained and analyzed for hormone profile assessment for the current study. The fertile control group included caucasians without vasectomy and had a child in the last five years with a sperm concentration and motility above the 50th percentile according to the parameters set forth in the 5th edition of the World Health Organization (WHO) guidelines in at least two spermiograms obtained after a 2–4 day period of sexual abstinence and with a 7-day period between tests. The hormones profiled used inclusion criteria of estradiol <50 pg/mL, FSH < 4.5 IU/L, total testosterone >300 ng/dL and bioavailable testosterone >145 ng/dL. Hormonal profiles were collected, dosed and analyzed following the clinical protocol in patients used in the current study.

Initial semen analysis and basal hormone determination to assess eligibility criteria were performed. Sperm samples were processed and stored for the subsequent epigenetic analysis. The infertility group received 150 IU of urinary or recombinant FSH three times per week for 12 weeks and the fertile control group did not received treatment. After three months of treatment, semen analysis and hormone profiles were retested in both groups. The sperm samples of three months with treatment for infertility and three months after for control group were processed and stored for the epigenetic test. Comité de Ética de la Investigación con medicamentos CEIM from the Health Research Institute Hospital La Fe (IIS La Fe), Institutional Review Board approval for the study was obtained, 2015-002521-19. The trial is identified in clinicaltrial.gov as NCT02605070.

### DNA preparation –

Performed as previously described^25^. Frozen human sperm samples were stored at −20 C and thawed for analysis. Genomic DNA from sperm was prepared as follows: A minimum of a one hundred μl of sperm suspension was used then 820 μl DNA extraction buffer (50 mM Tris pH 8, 10 mM EDTA pH 8, 0.5% SDS) and 80 μl 0.1 M Dithiothreitol (DTT) was added and the sample incubated at 65 C for 15 minutes. 80 μl Proteinase K (20 mg/ml) was added and the sample incubated on a rotator at 55 C for at least 2 hours. After incubation, 300 μl of protein precipitation solution (Promega, A795A, Madison, WI) was added, the sample was mixed and incubated on ice for 15 minutes, then spun at 4 C at 13,000 rpm for 30 minutes. The supernatant was transferred to a fresh tube, then precipitated over night at −20 C with the same volume 100% isopropanol and 2 μl glycoblue. The sample was then centrifuged and the pellet was washed with 75% ethanol, then air-dried and resuspended in 100 μl H_2_O. DNA concentration was measured using the Nanodrop (Thermo Fisher, Waltham, MA). The freeze-thaw will destroy any contaminating somatic cells within the sperm collection.

### Methylated DNA immunoprecipitation (MeDIP) –

Performed as previously described^25^. Methylated DNA Immunoprecipitation (MeDIP) with genomic DNA was performed as follows: individual sperm DNA samples were diluted to 130 μl with 1x Tris-EDTA (TE, 10 mM Tris, 1 mM EDTA) and sonicated with a the Covaris M220 using the 300 bp setting. Fragment size was verified on a 2% E-gel agarose gel. The sonicated DNA was transferred from the Covaris tube to a 1.7 ml microfuge tube and the volume was measured. The sonicated DNA was then diluted with TE buffer (10 mM Tris HCl, pH7.5; 1 mM EDTA) to 400 μl, heat-denatured for 10 min at 95 C, then immediately cooled on ice for 10 min. Then 100 μl of 5X IP buffer and 5 μg of antibody (monoclonal mouse anti 5-methyl cytidine; Diagenode #C15200006) were added to the denatured sonicated DNA. The DNA-antibody mixture was incubated overnight on a rotator at 4 C. The following day magnetic beads (Dynabeads M-280 Sheep anti-Mouse IgG; 11201D) were pre-washed as follows: The beads were resuspended in the vial, then the appropriate volume (50 μl per sample) was transferred to a microfuge tube. The same volume of Washing Buffer (at least 1 mL 1XPBS with 0.1% BSA and 2 mM EDTA) was added and the bead sample was resuspended. The tube was then placed into a magnetic rack for 1–2 minutes and the supernatant was discarded. The tube was removed from the magnetic rack and the beads were washed once. The washed beads were resuspended in the same volume of 1xIP buffer (50 mM sodium phosphate ph7.0, 700 mM NaCl, 0.25% TritonX-100) as the initial volume of beads. 50 μl of beads were added to the 500 μl of DNA-antibody mixture from the overnight incubation, then incubated for 2 h on a rotator at 4 C. After the incubation the bead-antibody-DNA complex was washed three times with 1X IP buffer as follows: The tube was placed into a magnetic rack for 1–2 minutes and the supernatant was discarded, then washed with 1xIP buffer 3 times. The washed bead-DNA solution was then resuspended in 250 μl digestion buffer with 3.5 μl Proteinase K (20 mg/ml). The sample was then incubated for 2–3 hours on a rotator at 55 C and then 250 μl of buffered Phenol-Chloroform- Isoamylalcohol solution was added to the sample and the tube was vortexed for 30 sec and then centrifuged at 14,000 rpm for 5 min at room temperature. The aqueous supernatant was carefully removed and transferred to a fresh microfuge tube. Then 250 μl chloroform were added to the supernatant from the previous step, vortexed for 30 sec and centrifuged at 14,000 rpm for 5 min at room temperature. The aqueous supernatant was removed and transferred to a fresh microfuge tube. To the supernatant 2 μl of glycoblue (20 mg/ml), 20 μl of 5 M NaCl and 500 μl ethanol were added and mixed well, then precipitated in −20 C freezer for 1 hour to overnight. The precipitate was centrifuged at 14,000 rpm for 20 min at 4 °C and the supernatant was removed, while not disturbing the pellet. The pellet was washed with 500 μl cold 70% ethanol in −20 C freezer for 15 min. then centrifuged again at 14,000 rpm for 5 min at 4 C and the supernatant was discarded. The tube was spun again briefly to collect residual ethanol to the bottom of the tube and as much liquid as possible was removed with gel loading tip. The pellet was air-dried at RT until it looked dry (about 5 minutes) then resuspended in 20 μl H2O or TE. DNA concentration was measured in Qubit (Life Technologies) with ssDNA kit (Molecular Probes Q10212).

### MeDIP-Seq analysis –

Performed as previously described^25^. The MeDIP DNA samples were used to create libraries for next generation sequencing (NGS) using the NEBNext Ultra RNA Library Prep Kit for Illumina (San Diego, CA) starting at step 1.4 of the manufacturer’s protocol to generate double stranded DNA. After this step the manufacturer’s protocol was followed. Each sample received a separate index primer. NGS was performed at WSU Spokane Genomics Core using the Illumina HiSeq 2500 with a PE50 application, with a read size of approximately 50 bp and approximately 20–25 million reads per sample and 9–10 sample libraries each were run in one lane.

### Molecular Bioinformatics and Statistics –

Performed as previously described^25^. Basic read quality was verified using summaries produced by the FastQC program^26^. Reads were filtered and trimmed to remove low quality base pairs using Trimmomatic^27^. Samples with elevated read depths were randomly subsampled to obtain more consistent read depths across all samples. The reads for each sample were mapped to the GRCh38 human genome using Bowtie2^28^ with default parameter options. The mapped read files were then converted to sorted BAM files using SAMtools^29^. To identify DMR, the reference genome was broken into 1000 bp windows. The MEDIPS R package^30^ was used to calculate differential coverage between control and exposure sample groups. The edgeR p-value^31^ was used to determine the relative difference between the two groups for each genomic window. Windows with an edgeR p-value less than 10^−5^ were considered DMRs. The DMR edges were extended until no genomic window with an edgeR p-value less than 0.1 remained within 1000 bp of the DMR. CpG density and other information was then calculated for the DMR based on the reference genome. DMR were annotated using the biomaRt R package^32^ to access the Ensembl database^33^. The genes that overlapped with DMR were then input into the KEGG pathway search^34,35^ to identify associated pathways. The DMR associated genes were then sorted into functional groups using information provided by the DAVID^36^ and Panther^37^ databases incorporated into an internal curated database (www.skinner.wsu.edu under genomic data). All MeDIP-Seq genomic data obtained in the current study have been deposited in the NCBI public GEO database (GEO #: GSE135825).

A permutation analysis to determine the significance of the number of DMR identified for each comparison was performed. For this analysis, samples from the two treatment groups were randomly assigned group membership. The number of samples in each treatment group was held constant. Twenty random permutations of each analysis were performed to obtain a null distribution for the expected number of DMR.

### Clinical statistical analysis

Performed as previously described^25^. In order to characterize clinical parameters of both groups (fertile and infertile group), a numerical descriptive analysis has been made using the mean with standard deviation (SD) and the median (1st and 3rd quartile). The baseline differences between the fertile group and the infertile group were then compared, as well as the effect of FSH between before and after treatment in the treated group, in all variables collected. For this, mixed linear regression models were used in case we had several measures per patient (semen volume and sperm concentration), and in the case of motility a beta logistic regression model was performed given its percentage character. The statistical mixed models controls for the non-independence of data given that there are several measures per patient.

In the fertile group both baseline and 3-month measures were considered, because no difference was expected. On the other hand, in the infertile treatment group, two samples were extracted from these three variables (volume, concentration and motility). In this way the power increases and there is a greater probability that we detect differences in case they exist. In all other cases, associations between variables have been studied using linear regression models.

The statistical analyses were performed with the statistical software R (version 3.4.1) and the packages nlme (version 3.1–131), lme4 (1.1–13), glmmADMB (0.8.3.3) and betareg (version 3.1–0). A p-value of less than 0.05 was considered statistically significant.

### List of abbreviations

Dithiothreitol (DTT)

DNA methylation regions (DMRs)

Follicle stimulating hormone (FSH)

Intracytoplasmic sperm injection (ICSI)

Methylated DNA Immunoprecipitation (MeDIP)

Next generation sequencing (NGS)

Principal component analysis (PCA)

Standard deviation (SD)

### Ethics approval and consent to participate

Comité de Ética de la Investigación con medicamentos CEIM from the Health Research Institute Hospital La Fe (IIS La Fe), Institutional Review Board approval for the study was obtained, 2015-002521-19. The trial is identified in clinicaltrial.gov as NCT02605070.

### Consent to participate

Informed consent to participate in this study was obtained from all participants.

### Declaration of helsinki

This study was carried out according to the Declaration of Helsinki.

## Supplementary information

Supplementary Information1

Supplementary Information2

Supplementary Information3

## Data Availability

All MeDIP-Seq genomic data obtained in the current study have been deposited in the NCBI public GEO database (GEO #: GSE135825).
