# Supplementary material for: Sperm DNA Methylation Epimutation Biomarkers for Male Infertility and FSH Therapeutic Responsiveness
Source: Sci Rep. 2019 Nov 14;9:16786. doi: 10.1038/s41598-019-52903-1 (PMC6856367; doi:10.1038/s41598-019-52903-1)
Supplement: Supplementary file 1 — Supplementary Information1 [file 41598_2019_52903_MOESM1_ESM.pdf]

Supplementary Table S1

A

Unblinded Patient ID	Selection Failure	Biomarker Use
**Fertile**		
Fer 010	no	yes
Fer 011	no	yes
Fer 012	no	yes
Fer 013	no	yes
Fer 017	no	yes
Fer 018	no	yes
Fer 020	no	yes
Fer 021	no	yes
Fer 027	no	yes
**Infertile**		
Inf 001	no	yes
Inf 002	no	yes
Inf 003	no	yes
Inf 005	no	yes
Inf 006	no	yes
Inf 007	no	yes
Inf 009	no	yes
Inf 012	no	yes
Inf 015	no	yes
Inf 016	no	yes
Inf 017	no	yes
Inf 018	no	yes
Inf 019	no	yes

B

Unblinded Patient ID	Selection Failure	Biomarker Use
**Samples with Selection Failure**		
**Fertile**		
Fer 006	yes	no
Fer 007	yes	no
Fer 008	yes	no
Fer 009	yes	no
Fer 014	yes	no
Fer 015	yes	no
Fer 016	yes	no
Fer 019	yes	no
Fer 022	yes	no
Fer 023	yes	no
Fer 024	yes	no
Fer Uncoded	yes	no
Fer Uncoded	yes	no
Fer Uncoded	yes	no
**Infertile**		
Inf 008	yes	no
Inf 020	yes	no
Inf 021	yes	no
Inf Uncoded	yes	no

C

	Pre-volume0	Pre-conc0	Pre-motile0	Pre-immotile0	Pre-TMC0
	SPERM UPON RECRUITMENT				
	Volsem0	Concsperm0	Motile0	Inmotile0	TMC
INF001	2.50	10.00	25.00	75.00	6.2
INF002	3.00	4.00	3.00	97.00	3.6
INF003	2.00	5.00	25.00	75.00	2.5
INF005	4.00	1.00	3.00	97.00	1.2
INF007	3.10	1.50	5.00	95.00	2.3
INF009	3.00	2.00	4.00	96.00	2.4
INF012	4.00	1.00	1.00	99.00	0.4
INF015	1.00	1.00	25.00	75.00	0.2
INF016	3.00	4.00	15.00	85.00	1.8
INF017	3.70	1.00	20.00	80.00	0.7
INF018	3.00	8.00	20.00	80.00	4.8
INF019	4.00	2.00	5.00	95.00	4

D

	Pre-volume1	Pre-conc1	Pre-motile1	Pre-immotile1	Pre-TMC1
	SPERM BEFORE THERAPY				
	VolSem1	Concsperm1	Motile1	Inmotile1	TMC
INF001	1	2	20	80	0.4
INF002	0.5	0.5	10	90	0.02
INF003	4	4	16	84	2.5
INF005	1.2	1.2	3.6	96.4	0.05
INF007	0.5	0.5	7	93	0.01
INF009	4	4	7.4	92.6	1.1
INF012	3	3	25	75	2.2
INF015	2	2	20	80	0.8
INF016	6	6	15	85	5.4
INF017	4	6	10	90	2.4
INF018	2	2	10	90	0.4
INF019	1	1	20	80	0.2

E

	Post-volume2	Post-conc2	Post-motile2	Post-immotile2	Post-TMC2
	SPERM AFTER THERAPY				
	Volsem2	Concsperm2	Motile2	Inmotile2	TMC
INF001	2	3	20	80	1.2
INF002	6	0.5	6.4	93.6	0.1
INF003	3	0.1	1	99	0.003
INF005	2	1.2	3.6	96.4	0.08
INF007	4	1	10	90	0.4
INF009	1.7	3	9.4	90.6	0.4
INF012	2.5	10	20	80	5
INF015	2	2	20	80	0.8
INF016	3	11	35	65	8.2
INF017	2	15	25	75	7.5
INF018	3	20	15	85	9
INF019	3	0.3	2	98	0.1
