# Supplementary material for: Sperm DNA Methylation Epimutation Biomarkers for Male Infertility and FSH Therapeutic Responsiveness
Source: Sci Rep. 2019 Nov 14;9:16786. doi: 10.1038/s41598-019-52903-1 (PMC6856367; doi:10.1038/s41598-019-52903-1)
Supplement: Supplementary file 2 — Supplementary Information2 [file 41598_2019_52903_MOESM2_ESM.pdf]

Supplementary Table S2
Fertility versus Infertility

DMR Name	Chr	Start	Stop	Length	# Sig Win	minP	maxLFC	CpG #	CpG Density	Gene Annotation	Gene Category
DMR1:629001	1	629001	635000	6000	4	1.10E-08	-1.3540969	159	2.65	AL669831.3;MTND1P23;MTND2P28;MTCO1P12;AC114498.2;MTCO2P12;MTATP8P1;MTATP6P1;MTCO3P12	
DMR1:4712001	1	4712001	4713000	1000	1	7.02E-06	-1.3546468	55	5.5	AJAP1	
DMR1:24099001	1	24099001	24101000	2000	1	5.74E-06	-1.3031799	64	3.2	MYOM3	Cytoskeleton
DMR1:121860001	1	121860001	121861000	1000	1	1.84E-06	-1.8326274	18	1.8		
DMR1:144475001	1	144475001	144476000	1000	1	2.11E-07	-1.3557747	24	2.4	AC246785.1	
DMR1:153464001	1	153464001	153465000	1000	1	5.26E-06	-1.2143061	14	1.4	S100A7	Signaling
DMR1:156732001	1	156732001	156733000	1000	1	1.55E-06	-1.3923279	10	1	ISG20L2;RRNAD1;MRPL24;HDGF	Transcription;Signaling
DMR1:204834001	1	204834001	204835000	1000	1	2.40E-06	0.9757061	10	1	NFASC	Extracellular Matrix
DMR1:226959001	1	226959001	226960000	1000	1	7.60E-06	-0.7873646	7	0.7	COQ8A;AL353689.1	
DMR2:3591001	2	3591001	3592000	1000	1	8.48E-06	-1.7009923	74	7.4	COLEC11	Immune
DMR2:10913001	2	10913001	10915000	2000	1	2.97E-06	-2.02485	83	4.15	KCNF1	Metabolism
DMR2:87091001	2	87091001	87092000	1000	1	3.41E-06	0.8659935	7	0.7		
DMR2:87348001	2	87348001	87350000	2000	1	1.87E-06	-1.2930736	162	8.1	IGKV3OR2-268	
DMR2:87405001	2	87405001	87432000	27000	6	1.36E-06	-1.7393104	237	0.878	LINC01943	
DMR2:101342001	2	101342001	101344000	2000	1	1.10E-07	-2.5359785	79	3.95	CREG2	
DMR2:104368001	2	104368001	104370000	2000	1	7.64E-06	-1.2924014	58	2.9		
DMR2:131646001	2	131646001	131647000	1000	1	3.57E-06	-1.2582149	47	4.7	LINC01087;GRAMD4P8	
DMR2:201858001	2	201858001	201859000	1000	1	1.94E-06	0.9091697	12	1.2	CDK15	Signaling
DMR2:211358001	2	211358001	211359000	1000	1	5.28E-06	0.931714	4	0.4		
DMR2:225582001	2	225582001	225583000	1000	1	2.80E-06	-2.2744394	71	7.1	NYAP2	
DMR3:112966001	3	112966001	112968000	2000	1	1.45E-07	0.7662919	6	0.3	CD200R1	Receptor
DMR3:125912001	3	125912001	125914000	2000	1	7.15E-06	-1.6588343	33	1.65	LINC02614;ENPP7P4;AC092903.2;FAM86JP	
DMR3:150778001	3	150778001	150779000	1000	1	8.51E-07	-1.6179423	27	2.7		
DMR3:176844001	3	176844001	176845000	1000	1	6.37E-09	1.003399	9	0.9	LINC01208	
DMR3:184170001	3	184170001	184171000	1000	1	5.67E-06	-2.1218771	69	6.9	DVL3;AP2M1	Signaling
DMR3:188433001	3	188433001	188434000	1000	1	1.84E-06	0.7498256	7	0.7	LPP	Cytoskeleton
DMR4:53001	4	53001	57000	4000	1	4.39E-07	-2.391171	136	3.4	BNIP3P41;ZNF595	Transcription
DMR4:747001	4	747001	749000	2000	1	8.83E-06	-1.9049842	122	6.1	PCGF3;AC139887.4	Transcription
DMR4:3809001	4	3809001	3811000	2000	1	1.96E-06	-2.285143	70	3.5		
DMR4:49270001	4	49270001	49274000	4000	1	1.80E-08	-1.4604032	100	2.5		
DMR4:49275001	4	49275001	49278000	3000	1	1.05E-07	-1.5488942	132	4.4		
DMR4:49279001	4	49279001	49284000	5000	1	9.37E-06	-1.721407	113	2.26		
DMR4:49291001	4	49291001	49303000	12000	2	7.79E-06	-1.5240589	279	2.325		
DMR4:49313001	4	49313001	49318000	5000	1	3.32E-06	-1.2186361	107	2.14		
DMR4:49319001	4	49319001	49325000	6000	1	4.35E-06	-1.4846204	132	2.2		
DMR4:49510001	4	49510001	49520000	10000	1	3.55E-06	-1.1945741	291	2.91	ANKRD20A17P;AC119751.5;AC119751.2;AC119751.8	
DMR4:68471001	4	68471001	68472000	1000	1	2.45E-06	1.361757	0	0	TMPRSS11E	Protease
DMR4:69599001	4	69599001	69600000	1000	1	4.86E-06	1.1306075	3	0.3	UGT2A1;UGT2A2	Metabolism
DMR4:185384001	4	185384001	185385000	1000	1	6.27E-06	1.0448613	10	1	LRP2BP;AC112722.1	Signaling
DMR4:186889001	4	186889001	186890000	1000	1	2.89E-06	-1.7304782	58	5.8	AC108865.1;AC108865.2	
DMR4:190021001	4	190021001	190023000	2000	1	8.41E-06	-0.9758405	169	8.45	RNA5SP174;RNA5SP175;DUX4L9;FRG2	
DMR5:258001	5	258001	262000	4000	1	1.75E-07	-2.7261411	343	8.575	SDHA;AC021087.5;AC021087.1;PDCD6;AHRR	Metabolism;Transcription
DMR5:1570001	5	1570001	1571000	1000	1	7.01E-06	-1.4101567	47	4.7	SDHAP3	
DMR5:12433001	5	12433001	12434000	1000	1	9.37E-06	0.8019226	3	0.3		
DMR5:17581001	5	17581001	17588000	7000	1	1.64E-06	-2.6252202	235	3.357	TAF11L7;AC233724.7;TAF11L8;TAF11L9;TAF11L10	
DMR5:17589001	5	17589001	17600000	11000	5	2.39E-06	-2.6544095	365	3.318	TAF11L7;AC233724.7;TAF11L8;TAF11L9;TAF11L10;AC233724.3;AC233724.6;TAF11L11	
DMR5:36783001	5	36783001	36784000	1000	1	1.90E-06	-1.5416817	35	3.5		
DMR5:46622001	5	46622001	46623000	1000	1	6.44E-06	-1.5676853	10	1		
DMR6:105831001	6	105831001	105832000	1000	1	2.05E-07	1.5144618	6	0.6	AL591518.1	
DMR6:132600001	6	132600001	132602000	2000	1	5.81E-06	1.5140375	4	0.2	TAAR4P;TAAR3P	
DMR6:150337001	6	150337001	150338000	1000	1	1.23E-06	-1.2839276	15	1.5		
DMR6:163194001	6	163194001	163197000	3000	1	4.93E-06	-2.1415117	64	2.133	PACRG;PACRG-AS3	Development
DMR6:167731001	6	167731001	167732000	1000	1	1.71E-07	-1.9579585	37	3.7		
DMR6:170138001	6	170138001	170139000	1000	1	6.23E-06	-1.4198044	43	4.3		
DMR7:636001	7	636001	638000	2000	1	4.76E-06	-2.1159539	119	5.95	PRKAR1B	Signaling
DMR7:2577001	7	2577001	2579000	2000	2	8.65E-07	-2.5038122	196	9.8	IQCE	

DMR7:10779001	7	10779001	10780000	1000	1	8.01E-07	-2.2960801	38	3.8	AC004949.1	
DMR7:37256001	7	37256001	37257000	1000	1	9.30E-07	1.0459778	6	0.6	ELMO1	Signaling
DMR7:58104001	7	58104001	58120000	16000	1	2.60E-07	-1.5731667	513	3.206		
DMR7:64407001	7	64407001	64408000	1000	1	3.09E-07	-1.5558383	31	3.1		
DMR7:85091001	7	85091001	85092000	1000	1	5.01E-06	0.9594642	7	0.7	SEMA3D	Growth Factors & Cytokines
DMR7:128586001	7	128586001	128587000	1000	1	9.46E-06	-0.8221203	7	0.7	AC090114.3;AC108010.1	
DMR7:155788001	7	155788001	155790000	2000	1	4.97E-06	-1.6163775	105	5.25	RBM33;SHH	Signaling
DMR8:12541001	8	12541001	12542000	1000	1	2.30E-06	-1.2106429	18	1.8	AC068587.4	
DMR8:23248001	8	23248001	23249000	1000	1	9.44E-06	-1.3344564	19	1.9	CHMP7	Binding Protein
DMR8:42053001	8	42053001	42054000	1000	1	6.30E-08	-1.9852231	31	3.1	KAT6A;RF01169	Transcription
DMR8:46103001	8	46103001	46106000	3000	1	1.92E-06	-1.1795439	102	3.4		
DMR8:46257001	8	46257001	46261000	4000	1	1.70E-07	-1.7683408	111	2.775		
DMR8:52388001	8	52388001	52389000	1000	1	8.83E-06	-1.8314409	30	3	ST18	Transcription
DMR8:85642001	8	85642001	85644000	2000	1	5.21E-06	-2.3394436	118	5.9	REXO1L8P	
DMR8:85645001	8	85645001	85664000	19000	4	1.93E-07	-2.464287	566	2.979	REXO1L8P;REXO1L3P;REXO1L1P	
DMR8:85714001	8	85714001	85766000	52000	12	1.17E-08	-3.0057258	1357	2.61	REXO1L12P;REXO1L11P;REXO1L10P;REXO1L9P;REXO1L2P	
DMR8:85767001	8	85767001	85781000	14000	2	2.49E-07	-2.6820478	361	2.579	REXO1L9P;REXO1L2P;AC232323.1	
DMR8:85782001	8	85782001	85830000	48000	13	3.62E-09	-2.8895738	1277	2.66	REXO1L2P;AC232323.1;REXO1L4P;REXO1L5P;REXO1L6P;AC100801.1	
DMR8:112511001	8	112511001	112512000	1000	1	1.79E-06	-1.1234341	23	2.3	CSMD3	
DMR8:133772001	8	133772001	133774000	2000	1	1.84E-06	-1.0463593	36	1.8	AC133634.1;AC090821.1	
DMR9:3536001	9	3536001	3537000	1000	1	4.61E-06	-1.5886032	3	0.3	RFX3;RFX3-AS1	Transcription
DMR9:19129001	9	19129001	19130000	1000	1	6.66E-06	-1.5906467	25	2.5	PLIN2	
DMR9:41235001	9	41235001	41236000	1000	1	5.63E-07	-1.1919131	57	5.7	MIR4477A;RNA5SP530	
DMR9:41644001	9	41644001	41646000	2000	1	2.98E-06	-1.8468202	79	3.95	AL591926.6;AL591926.5;AL591926.2	
DMR9:43111001	9	43111001	43112000	1000	1	1.48E-07	-1.746655	29	2.9	FP325317.1	
DMR9:61669001	9	61669001	61670000	1000	1	9.79E-06	-1.6439159	40	4	AL935212.1;AL935212.2	
DMR9:113083001	9	113083001	113086000	3000	1	1.68E-06	-3.0084045	156	5.2	AL449105.4;AL449105.2;AL449105.5	
DMR9:125425001	9	125425001	125428000	3000	1	6.86E-06	-0.8153035	88	2.933	RF00017;MAPKAP1	Signaling
DMR9:137809001	9	137809001	137811000	2000	1	1.63E-06	-1.6804834	112	5.6	EHMT1	Transcription
DMR10:5512001	10	5512001	5514000	2000	1	4.05E-06	-2.761433	64	3.2	CALML3-AS1	
DMR10:18917001	10	18917001	18919000	2000	1	1.91E-06	-1.0126471	36	1.8		
DMR10:29254001	10	29254001	29255000	1000	1	6.67E-06	1.2016721	9	0.9		
DMR10:32286001	10	32286001	32287000	1000	1	4.78E-06	-1.1833967	12	1.2	EPC1;AL158834.1	Metabolism
DMR10:40846001	10	40846001	40847000	1000	1	2.02E-08	-1.7704581	15	1.5		
DMR10:45786001	10	45786001	45787000	1000	1	7.22E-06	-0.7814647	18	1.8	WASHC2C	
DMR10:67563001	10	67563001	67564000	1000	1	1.83E-06	-1.003488	4	0.4	CTNNA3	Cytoskeleton
DMR10:76034001	10	76034001	76036000	2000	1	2.93E-06	-0.8510863	46	2.3	LRMDA	
DMR10:125192001	10	125192001	125194000	2000	1	2.87E-06	-0.8868839	47	2.35		
DMR10:125896001	10	125896001	125899000	3000	1	9.30E-06	-1.9587022	160	5.333	DHX32;RNU2-42P;FANK1	Transcription
DMR10:132443001	10	132443001	132445000	2000	1	2.13E-06	1.6143491	44	2.2	AL451069.3;C10orf91	
DMR10:132858001	10	132858001	132860000	2000	1	4.83E-06	-1.494898	85	4.25	CFAP46	
DMR11:33429001	11	33429001	33431000	2000	1	4.80E-08	0.9745725	24	1.2	KIAA1549L	
DMR11:64607001	11	64607001	64608000	1000	1	9.76E-06	-1.5046308	73	7.3	SLC22A12;NRXN2	Transport;Receptor
DMR11:71593001	11	71593001	71594000	1000	1	3.91E-06	-1.3475282	19	1.9	KRTAP5-11;OR7E87P;UNC93B6	
DMR11:134288001	11	134288001	134289000	1000	1	9.06E-06	-1.8607813	22	2.2	GLB1L3	Golgi
DMR12:1638001	12	1638001	1640000	2000	1	3.50E-06	-1.9194063	97	4.85	WNT5B	Signaling
DMR12:10027001	12	10027001	10028000	1000	1	4.47E-06	0.8480909	5	0.5	CLEC12B;AC024224.2;CLEC9A	
DMR12:22036001	12	22036001	22037000	1000	1	5.92E-06	-1.0274171	21	2.1	CMAS	Metabolism
DMR12:35064001	12	35064001	35065000	1000	1	2.24E-06	-1.5044425	17	1.7		
DMR12:54195001	12	54195001	54197000	2000	1	6.68E-06	0.8359955	28	1.4	SMUG1	Transcription
DMR12:56500001	12	56500001	56501000	1000	1	9.18E-06	-0.9025905	30	3		
DMR12:56595001	12	56595001	56596000	1000	1	2.90E-06	-1.3567206	10	1	RBMS2;RNU6-343P;BAZ2A	Translation;Transcription
DMR12:117606001	12	117606001	117607000	1000	1	9.33E-06	1.4461211	4	0.4	KSR2	Signaling
DMR12:124208001	12	124208001	124209000	1000	1	9.81E-06	-2.2382942	27	2.7	RFLNA;AC026358.1	
DMR12:131087001	12	131087001	131089000	2000	1	9.95E-06	-2.0205843	85	4.25	ADGRD1	
DMR13:23084001	13	23084001	23086000	2000	1	1.30E-06	-1.2929502	50	2.5		
DMR13:30392001	13	30392001	30393000	1000	1	4.82E-06	-0.8768676	14	1.4	AL161893.1	
DMR13:57140001	13	57140001	57144000	4000	1	3.02E-06	-2.431105	144	3.6	PRR20A;PRR20C;PRR20B	
DMR13:57146001	13	57146001	57151000	5000	3	9.83E-07	-2.3210882	183	3.66	PRR20A;PRR20C;PRR20B;PRR20D	
DMR13:57152001	13	57152001	57157000	5000	1	5.11E-06	-2.4181847	184	3.68	PRR20A;PRR20C;PRR20B;PRR20D	
DMR13:57165001	13	57165001	57171000	6000	2	3.23E-07	-2.8351351	204	3.4	PRR20C;PRR20D;PRR20E;PRR20FP	
DMR13:57172001	13	57172001	57174000	2000	1	5.17E-06	-2.5067514	80	4	PRR20D;PRR20E;PRR20FP	
DMR13:76849001	13	76849001	76850000	1000	1	3.96E-06	-1.5185848	19	1.9	AL136441.1;AL365394.1	
DMR13:113834001	13	113834001	113835000	1000	1	1.98E-06	-1.5471945	52	5.2	GAS6-AS1;GAS6	Signaling
DMR14:19337001	14	19337001	19340000	3000	1	4.91E-06	-1.7315727	80	2.667	AL589743.1;LINC01297	

DMR14:19361001	14	19361001	19363000	2000	1	2.26E-08	-1.9640587	106	5.3	LINC01297;GRAMD4P3	
DMR14:19678001	14	19678001	19680000	2000	1	3.12E-06	-2.2198228	56	2.8	AL512310.11;AL512310.4;AL512310.5;AL512310.6;AL512310.9;AL512310.7;AL512310.2;ARHGAP42P4	
DMR14:70233001	14	70233001	70235000	2000	1	3.03E-06	-1.956227	70	3.5	AL160191.1;AL160191.3;AL160191.2	
DMR15:20799001	15	20799001	20801000	2000	1	1.10E-06	-2.2910423	104	5.2	AC012414.7	
DMR15:25389001	15	25389001	25391000	2000	1	8.18E-07	-1.0766537	14	0.7	SNHG14;UBE3A	Metabolism
DMR15:31442001	15	31442001	31443000	1000	1	7.44E-06	-1.7221919	21	2.1	KLF13	Transcription
DMR15:47155001	15	47155001	47156000	1000	1	6.14E-08	-1.5006382	22	2.2		
DMR16:1092001	16	1092001	1096000	4000	2	2.94E-06	-1.2310444	216	5.4	C1QTNF8;AL031713.1	Immune
DMR16:2750001	16	2750001	2752000	2000	1	2.41E-06	-1.8140343	59	2.95	SRRM2-AS1;SRRM2	
DMR16:13235001	16	13235001	13236000	1000	1	4.52E-07	-1.5067152	32	3.2	SHISA9	
DMR16:34571001	16	34571001	34577000	6000	4	8.99E-07	-1.243301	200	3.333		
DMR16:34580001	16	34580001	34602000	22000	4	9.45E-07	-1.2723349	759	3.45		
DMR16:34603001	16	34603001	34612000	9000	1	1.71E-06	-1.5120863	341	3.789		
DMR16:34717001	16	34717001	34729000	12000	1	8.96E-06	-1.543084	432	3.6		
DMR16:34946001	16	34946001	34961000	15000	1	5.80E-06	-1.4539215	547	3.647	AC135776.4	
DMR16:46380001	16	46380001	46423000	43000	8	9.08E-07	-1.2446121	1611	3.747		
DMR16:74985001	16	74985001	74986000	1000	1	2.38E-06	-1.5455224	61	6.1	WDR59	
DMR16:81051001	16	81051001	81052000	1000	1	6.78E-06	-1.0652411	7	0.7	AC092718.8;ATMIN;C16orf46;AC092718.3;AC092718.5	DNA Repair
DMR16:86681001	16	86681001	86684000	3000	1	1.91E-07	-1.639672	85	2.833		
DMR16:88184001	16	88184001	88186000	2000	1	7.72E-08	-2.1978318	77	3.85	AC134312.2;AC134312.5;AC134312.6;LINC02182	
DMR16:88531001	16	88531001	88533000	2000	1	8.62E-07	-2.0612887	83	4.15	ZFPM1;AC116552.1	Transcription
DMR16:89281001	16	89281001	89282000	1000	1	5.15E-06	-1.5758789	66	6.6	ANKRD11;AC137932.3	EST
DMR17:2692001	17	2692001	2693000	1000	1	4.59E-06	-2.0005593	76	7.6	PAFAH1B1;AC005696.2;AC005696.3;CLUH;MIR6776	Metabolism
DMR17:8227001	17	8227001	8229000	2000	1	3.68E-06	-1.291337	38	1.9	LINC00324;CTC1	
DMR17:24421001	17	24421001	24422000	1000	1	6.34E-07	-1.6340753	19	1.9		
DMR17:25074001	17	25074001	25075000	1000	1	2.20E-06	-1.8468259	26	2.6		
DMR17:26625001	17	26625001	26627000	2000	1	4.27E-07	-1.9925314	34	1.7		
DMR17:26881001	17	26881001	26886000	5000	2	5.76E-06	-1.4743264	68	1.36		
DMR17:31561001	17	31561001	31562000	1000	1	6.61E-07	-1.2921192	13	1.3	MIR193A;AC003101.2;RNU6ATAC7P;AC003101.1	
DMR17:42404001	17	42404001	42406000	2000	1	9.49E-07	-1.8382718	141	7.05	CAVIN1	
DMR17:50220001	17	50220001	50222000	2000	1	1.21E-06	-1.6834102	44	2.2	AC015909.1;AC015909.4	
DMR17:80661001	17	80661001	80662000	1000	1	1.66E-06	-1.0482083	33	3.3	RPTOR	
DMR17:82359001	17	82359001	82362000	3000	1	4.41E-06	1.1770702	10	0.333	TEX19;AC132938.4	
DMR18:9868001	18	9868001	9869000	1000	1	6.69E-06	-0.8901627	21	2.1	RAB31	Signaling
DMR18:12375001	18	12375001	12376000	1000	1	5.16E-06	-1.8792193	43	4.3	AFG3L2	Protease
DMR18:14488001	18	14488001	14490000	2000	1	6.26E-06	-1.582541	92	4.6	CXADRP3;GRAMD4P7	
DMR18:20578001	18	20578001	20580000	2000	1	4.60E-06	-1.3916313	38	1.9		
DMR18:40983001	18	40983001	40985000	2000	1	4.12E-06	0.8892648	9	0.45		
DMR18:76611001	18	76611001	76613000	2000	2	8.41E-07	-2.0375198	85	4.25	LINC00683;AC034110.1	
DMR19:9232001	19	9232001	9234000	2000	1	3.12E-06	-1.2938746	43	2.15	OR7D1P	
DMR19:15456001	19	15456001	15457000	1000	1	4.17E-06	-1.7724757	31	3.1	WIZ;MIR1470;RASAL3	
DMR19:29638001	19	29638001	29641000	3000	1	1.08E-06	-1.6066412	78	2.6		
DMR19:36273001	19	36273001	36310000	37000	5	1.07E-07	-2.8235712	2586	6.989	AC012617.1;LINC00665	
DMR19:37269001	19	37269001	37304000	35000	3	1.06E-06	-2.5754265	2445	6.986	AC016590.1;LINC01535;HKR1	Transcription
DMR19:39482001	19	39482001	39483000	1000	1	7.68E-06	-1.0961949	27	2.7	SUPT5H;TIMM50	Transcription;Metabolism
DMR19:45474001	19	45474001	45475000	1000	1	1.19E-06	-1.5692376	37	3.7	ERCC1;FOSB	Epigenetic;Transcription
DMR19:49209001	19	49209001	49212000	3000	1	5.35E-06	-1.0071734	94	3.133	TRPM4	Development
DMR19:49862001	19	49862001	49863000	1000	1	4.63E-07	-2.1268905	63	6.3	PTOV1;AC018766.1;MIR4749;PTOV1-AS2;PNKP;AKT1S1	Development;DNA Repair
DMR19:50908001	19	50908001	50909000	1000	1	4.46E-06	-1.0203746	36	3.6	KLK4	Protease
DMR19:53768001	19	53768001	53769000	1000	1	9.73E-06	-1.1968045	52	5.2	MIR1283-2;RNU6-1041P;MIR516A2;AC011453.1;MIR519A2;RNU6-165P;HMGN1P32;SEPT7P8;AC008753.1	
DMR19:55616001	19	55616001	55617000	1000	1	4.94E-06	-1.6534294	90	9	ZNF865;AC008735.4;ZNF784	Transcription
DMR19:56197001	19	56197001	56198000	1000	1	1.38E-07	-2.5628136	50	5	ZSCAN5B;ZSCAN5C	Transcription
DMR20:419001	20	419001	422000	3000	1	8.84E-08	-2.3117104	121	4.033	RBCK1	Metabolism
DMR20:5069001	20	5069001	5070000	1000	1	2.76E-06	0.8082335	11	1.1	AL121890.5;AL121890.4;TMEM230	
DMR20:18092001	20	18092001	18094000	2000	1	6.51E-06	-1.8216619	59	2.95	RPL15P1;RNU7-137P	
DMR20:23365001	20	23365001	23366000	1000	1	2.90E-06	-1.7760581	67	6.7	LINC01431;GZF1;NAPB	Transcription
DMR20:26696001	20	26696001	26697000	1000	1	3.59E-06	-1.9464181	18	1.8		
DMR20:27990001	20	27990001	27991000	1000	1	4.21E-06	-1.905119	17	1.7		
DMR20:63970001	20	63970001	63971000	1000	1	9.14E-06	1.3517273	46	4.6	ZNF512B;SAMD10	Transcription

DMR21:8806001	21	8806001	8816000	10000	2	1.42E-07	-1.6852815	317	3.17	CR381670.2;SNX18P10	
DMR21:9067001	21	9067001	9071000	4000	2	4.72E-06	-1.527546	110	2.75	CR392039.5;TEKT4P2	
DMR21:9086001	21	9086001	9089000	3000	1	3.73E-06	-1.1750689	125	4.167	TEKT4P2;CR392039.4;CR392039.1	
DMR21:9872001	21	9872001	9874000	2000	1	4.96E-06	-0.9015005	18	0.9		
DMR21:12679001	21	12679001	12680000	1000	1	3.89E-06	-1.9111383	18	1.8		
DMR21:20781001	21	20781001	20783000	2000	1	3.21E-06	-1.8885383	39	1.95	LINC00320	
DMR21:32277001	21	32277001	32278000	1000	1	6.65E-06	-1.5881648	28	2.8	MIS18A;MIS18A-AS1	
DMR21:37919001	21	37919001	37920000	1000	1	4.79E-06	-1.4685089	12	1.2	KCNJ6	Metabolism
DMR21:46007001	21	46007001	46008000	1000	1	3.56E-06	-1.0562453	25	2.5	COL6A1	Cytoskeleton
DMR22:10576001	22	10576001	10577000	1000	1	6.16E-08	-1.5166733	10	1		
DMR22:10703001	22	10703001	10704000	1000	1	4.84E-06	-1.7196769	6	0.6		
DMR22:10738001	22	10738001	10740000	2000	1	6.36E-06	-1.0923415	32	1.6	RF00004	
DMR22:10741001	22	10741001	10745000	4000	1	4.03E-06	-1.758626	162	4.05	RF00004	
DMR22:11609001	22	11609001	11612000	3000	1	1.54E-06	-0.8957715	77	2.567		
DMR22:12167001	22	12167001	12179000	12000	1	1.50E-06	-1.2108072	342	2.85		
DMR22:15563001	22	15563001	15564000	1000	1	5.86E-06	-1.8164072	26	2.6	AP000534.2;ARHGAP42P3;AP000534.1;AP000533.2;AP000533.1	
DMR22:15572001	22	15572001	15574000	2000	1	2.20E-06	-1.4760505	79	3.95	AP000534.2;AP000533.2;AP000533.1	
DMR22:16305001	22	16305001	16343000	38000	1	8.43E-06	-1.3536179	1370	3.605		
DMR22:18845001	22	18845001	18847000	2000	1	9.41E-07	-1.7097476	87	4.35	GGT3P;AC008132.1;BCRP7	
DMR22:24250001	22	24250001	24252000	2000	1	7.29E-06	-1.6278471	82	4.1	GGT5;GGTLC4P;POM121L9P;BCRP1	Metabolism
DMR22:24454001	22	24454001	24455000	1000	1	1.49E-06	-0.8764264	13	1.3	ADORA2A-AS1	
DMR22:34157001	22	34157001	34159000	2000	1	5.97E-07	-1.2803132	41	2.05	LINC01643	
DMR22:37444001	22	37444001	37446000	2000	1	8.88E-08	-0.8579501	41	2.05		
DMR22:48286001	22	48286001	48287000	1000	1	5.03E-08	-1.7535225	15	1.5		
DMRMT:1	MT	1	16569	16569	17	3.41E-10	-2.3369179	435	2.625	MT-TF;MT-RNR1;MT-TV;MT-RNR2;MT-TL1;MT-ND1;MT-TI;MT-TQ;MT-TM;MT-ND2;MT-TW;MT-TA;MT-TN;MT-TC;MT-TY;MT-CO1;MT-TS1;MT-TD;MT-CO2;MT-TK;MT-ATP8;MT-ATP6;MT-CO3;MT-TG;MT-ND3;MT-TR;MT-ND4L;MT-ND4;MT-TH;MT-TS2;MT-TL2;MT-ND5;MT-ND6;MT-TE;MT-CYB;MT-TT;MT-TP	
DMRX:268001	X	268001	271000	3000	1	9.96E-07	-1.7180105	136	4.533	PLCXD1	
DMRX:10094001	X	10094001	10095000	1000	1	3.07E-07	1.0653938	17	1.7	WWC3	
DMRX:49339001	X	49339001	49342000	3000	1	9.07E-06	-1.178808	113	3.767	GAGE12J;GAGE13;GAGE2E	
DMRX:49589001	X	49589001	49591000	2000	1	3.70E-06	-2.2773116	87	4.35	GAGE12H;GAGE1;GAGE2A	
DMRX:56594001	X	56594001	56595000	1000	1	1.85E-06	-1.2924059	10	1		
DMRX:140977001	X	140977001	140978000	1000	1	5.52E-06	0.9405132	9	0.9	AL451048.1	
DMRY:6246001	Y	6246001	6247000	1000	1	1.67E-07	-2.27506	64	6.4	TTTY23B;TSPY2;FAM197Y9	Protein Binding
DMRY:6265001	Y	6265001	6266000	1000	1	6.09E-06	-2.3232095	55	5.5	FAM197Y9;TSPY11P;AC006335.2	
DMRY:9356001	Y	9356001	9357000	1000	1	3.09E-06	-2.5246157	59	5.9	FAM197Y8;TSPY8	Protein Binding
DMRY:9395001	Y	9395001	9400000	5000	1	6.16E-06	-2.3246137	204	4.08	FAM197Y6;AC006158.3;TSPY3	Protein Binding
DMRY:9505001	Y	9505001	9509000	4000	1	7.26E-07	-2.3500189	188	4.7	FAM197Y3;TSPY6P;FAM197Y2	
DMRY:21896001	Y	21896001	21897000	1000	1	6.37E-06	-2.1121986	30	3	RBMY1D;AC007322.4;RBMY1E	Transcription
