# Supplementary material for: Sperm DNA Methylation Epimutation Biomarkers for Male Infertility and FSH Therapeutic Responsiveness
Source: Sci Rep. 2019 Nov 14;9:16786. doi: 10.1038/s41598-019-52903-1 (PMC6856367; doi:10.1038/s41598-019-52903-1)
Supplement: Supplementary file 3 — Supplementary Information3 [file 41598_2019_52903_MOESM3_ESM.pdf]

Supplementary Table S3
Non-Responder versus Responder

DMR Name	Chr	Start	Stop	Length	# Sig Win	minP	maxLFC	CpG #	CpG Density	Gene Annotation	Gene Category
DMR1:17235001	1	17235001	17236000	1000	1	9.11E-07	1.0645622	12	1.2	PADI1	Metabolism
DMR1:25292001	1	25292001	25294000	2000	1	9.84E-09	-2.1663744	11	0.55	RSRP1;RHD;SDHDP6	Transport
DMR1:25299001	1	25299001	25300000	1000	1	2.76E-09	-1.8483392	23	2.3	RSRP1;RHD;SDHDP6	Transport
DMR1:25304001	1	25304001	25305000	1000	1	8.29E-06	-1.8151307	19	1.9	RSRP1;RHD;SDHDP6	Transport
DMR1:25318001	1	25318001	25319000	1000	1	1.70E-07	-2.0585241	15	1.5	RSRP1;RHD	Transport
DMR1:25333001	1	25333001	25334000	1000	1	2.49E-06	-1.3360508	16	1.6	RSRP1;RHD;AL928711.1;TMEM50A	Transport
DMR1:218548001	1	218548001	218549000	1000	1	7.63E-06	-1.2569819	13	1.3	RF00012	
DMR2:163904001	2	163904001	163905000	1000	1	6.54E-06	-1.4222477	4	0.4	AC016766.1	
DMR3:9159001	3	9159001	9160000	1000	1	7.15E-06	-1.0760625	13	1.3	SRGAP3	Signaling
DMR3:18212001	3	18212001	18213000	1000	1	1.06E-06	-1.4381627	5	0.5	TBC1D5	Signaling
DMR3:25359001	3	25359001	25360000	1000	1	8.09E-06	-1.2918398	3	0.3	RARB;RNA5SP126	Signaling
DMR3:36185001	3	36185001	36186000	1000	1	1.81E-07	-1.3766023	5	0.5		
DMR3:111089001	3	111089001	111090000	1000	1	8.67E-07	-1.5806562	8	0.8	NECTIN3	
DMR3:118291001	3	118291001	118293000	2000	1	4.57E-08	-1.4412971	12	0.6	AC068633.1	
DMR3:120974001	3	120974001	120977000	3000	1	5.90E-06	-1.2434688	20	0.667	STXBP5L	Transcription
DMR4:164482001	4	164482001	164483000	1000	1	2.00E-06	1.3078442	4	0.4		
DMR4:170150001	4	170150001	170151000	1000	1	4.27E-07	-1.5550518	4	0.4	AC069306.1	
DMR4:187047001	4	187047001	187048000	1000	1	8.29E-06	1.1028096	16	1.6	AC110772.2	
DMR5:15460001	5	15460001	15461000	1000	1	5.64E-06	-0.9900734	6	0.6	AC114964.1	
DMR5:20858001	5	20858001	20859000	1000	1	8.86E-06	-1.1168998	7	0.7	LINC02241	
DMR5:84058001	5	84058001	84059000	1000	1	3.43E-06	-1.3418608	7	0.7	EDIL3	Extracellular Matrix
DMR5:113458001	5	113458001	113460000	2000	1	6.47E-06	1.1312994	31	1.55	MCC	Transcription
DMR5:122270001	5	122270001	122271000	1000	1	8.74E-07	-1.3435475	4	0.4		
DMR6:5284001	6	5284001	5285000	1000	1	2.64E-06	-1.2871156	11	1.1	FARS2;AL121978.1	
DMR6:169552001	6	169552001	169554000	2000	1	3.18E-06	1.5327536	42	2.1	AL031315.1;WDR27	
DMR7:69279001	7	69279001	69280000	1000	1	4.90E-06	1.0949466	11	1.1	AC092100.1	
DMR7:119280001	7	119280001	119281000	1000	1	3.05E-07	-1.1363468	5	0.5		
DMR8:10912001	8	10912001	10913000	1000	1	1.65E-06	-1.6874264	7	0.7	XKR6	Immune
DMR8:55562001	8	55562001	55563000	1000	1	7.25E-06	-1.2043517	4	0.4		
DMR9:17319001	9	17319001	17320000	1000	1	9.15E-06	-1.3272344	5	0.5	CNTLN	
DMR9:22122001	9	22122001	22123000	1000	1	1.03E-07	-1.5186233	5	0.5	CDKN2B-AS1;RF01909	
DMR9:65745001	9	65745001	65746000	1000	1	6.00E-06	-1.2615133	7	0.7	FOXD4L4	
DMR9:89370001	9	89370001	89374000	4000	1	9.89E-06	1.2449168	56	1.4	SEMA4D	Development
DMR9:124203001	9	124203001	124204000	1000	1	2.36E-06	1.1490304	18	1.8		
DMR9:130473001	9	130473001	130474000	1000	1	1.19E-06	1.6118581	23	2.3	ASS1	Development
DMR9:134476001	9	134476001	134477000	1000	1	6.96E-08	-1.300523	12	1.2		
DMR10:559001	10	559001	560000	1000	1	4.86E-06	1.0297783	21	2.1	DIP2C	
DMR10:3718001	10	3718001	3719000	1000	1	1.33E-06	1.5500791	16	1.6		
DMR10:7136001	10	7136001	7137000	1000	1	9.63E-07	-1.6184753	14	1.4		
DMR10:36569001	10	36569001	36570000	1000	1	1.59E-07	-1.4504064	20	2		
DMR11:19552001	11	19552001	19553000	1000	1	1.12E-06	1.327054	18	1.8	NAV2	Development
DMR11:126188001	11	126188001	126189000	1000	1	1.92E-06	0.991244	21	2.1	AP001893.2	
DMR12:62236001	12	62236001	62238000	2000	1	6.81E-06	-1.1166275	22	1.1	FAM19A2;KLF17P1	Growth Factors & Cytokines
DMR12:121750001	12	121750001	121752000	2000	1	9.27E-06	1.3819881	18	0.9	TMEM120B	Unknown
DMR12:131647001	12	131647001	131648000	1000	1	7.17E-06	1.084876	23	2.3	AC117500.4;LINC02414	
DMR13:37820001	13	37820001	37821000	1000	1	8.53E-08	-1.8339284	4	0.4	TRPC4	Development
DMR14:81859001	14	81859001	81860000	1000	1	1.86E-06	-1.3402382	14	1.4	AL355838.1	
DMR16:23408001	16	23408001	23409000	1000	1	2.13E-06	1.2552037	34	3.4	COG7;RN7SKP23	Golgi
DMR16:28375001	16	28375001	28376000	1000	1	1.44E-06	1.3194317	13	1.3	AC138894.3;EIF3CL	Translation
DMR17:45915001	17	45915001	45916000	1000	1	8.73E-06	0.8078421	12	1.2	MAPT;CR936218.2	Cytoskeleton
DMR17:46235001	17	46235001	46236000	1000	1	4.74E-06	1.0572367	13	1.3	KANSL1;MAPK8IP1P1	
DMR17:46297001	17	46297001	46298000	1000	1	5.15E-06	0.9689937	11	1.1	ARL17B;LRRC37A	Signaling
DMR17:73426001	17	73426001	73428000	2000	1	4.27E-06	-1.3244611	24	1.2	SDK2	Development
DMR19:2712001	19	2712001	2715000	3000	1	3.78E-06	1.1579408	43	1.433	GNG7;DIRAS1;AC006538.2	Signaling
DMR20:29885001	20	29885001	29886000	1000	1	2.32E-06	1.3647393	5	0.5	DUX4L37	
DMRX:119779001	X	119779001	119780000	1000	1	7.18E-06	1.161398	21	2.1	SNORA69;RPL39	Translation
